# Clinicopathological predictive factors for ipsilateral and contralateral events following initial surgery to treat ductal carcinoma in situ

**DOI:** 10.1007/s12282-015-0595-x

**Published:** 2015-02-11

**Authors:** Nobuko Tamura, Hitoshi Tsuda, Masayuki Yoshida, Takashi Hojo, Sadako Akashi-Tanaka, Takayuki Kinoshita, Kenichi Sugihara

**Affiliations:** 1 Department of Breast and Endocrine Surgery, Toranomon Hospital, Tokyo, Japan; 2 Department of Pathology and Clinical Laboratories, National Cancer Center Hospital, Tokyo, Japan; 3 Breast Surgery Division, National Cancer Center Hospital, Tokyo, Japan; 4 Division of breast Surgical Oncology, Showa University School of Medicine, Tokyo, Japan; 5 Department of Basic Pathology, National Defense Medical College, Namiki 3-2, Tokorozawa, Saitama 359-8513 Japan; 6 Surgical Oncology Division, Tokyo Medical and Dental University, Tokyo, Japan

**Keywords:** Ductal carcinoma in situ (DCIS), Ispilateral breast tumor recurrence (IBTR), Contralateral breast tumor recurrence (CBTR), Histological subtype, Intrinsic subtype, Nuclear grade

## Abstract

**Background:**

Ipsilateral breast tumor recurrence (IBTR) after partial breast resection and contralateral breast tumor recurrence (CBTR) have been shown to occur relatively frequently in patients with ductal carcinoma in situ (DCIS). However, there is only limited data from Japanese institutes to support this.

**Methods:**

Of 301 consecutive DCIS patients, 179 patients underwent a mastectomy, and the other 122 underwent partial resection in the National Cancer Center Hospital, Tokyo, with a median follow-up period of 2,106 days. We reviewed clinicopathological parameters including age, menopausal status, body mass index, family history (FH) of breast cancer, tumor size, histological subtype, nuclear grade (NG), hormone receptor (HR) and human epidermal growth factor receptor 2 (HER2) status, treatment, and the surgical margin status of partially resected specimens. The risk associated with each of these parameters for IBTR in 122 patients who underwent partial resections, and for CBTR in a total of 301 patients were calculated using Cox proportional hazard general linear models.

**Results:**

Of the 122 patients who underwent partial breast resection, IBTR occurred in 7 (5.7 %). The risk of IBTR was higher or tended to be higher in younger patients or those with lower NG tumors, but did not change significantly with respect to margin status or irradiation. Amongst the entire cohort of 301 patients, CBTR occurred in 18 cases (6.0 %). CBTR occurred significantly more frequently in patients with a FH of breast cancer and with HR+/HER2− subtype tumors by univariate analyses, and tumor subtype was an independent risk factor for CBTR by multivariate analysis.

**Conclusions:**

The local recurrence rate was low following partial resection of DCIS. Younger age was a risk factor for IBTR, whereas the HR+/HER2− tumor subtype and a FH of breast cancer were risk factors for CBTR.

## Introduction

The proportion of ductal carcinoma in situ (DCIS) amongst all surgically resected breast cancers is reported to be 20 % in Western countries and nearly 10 % in Japan [1–5]. Pure DCIS in itself is not a life threatening disease, and the local recurrences if that appear as DCIS do not influence the overall survival rate of patients. True DCIS will theoretically not metastasize to regional lymph nodes or relapse in a distant organ, and thus the management of DCIS patients focuses on local control of the primary lesion and early detection and treatment of both ispilateral breast tumor recurrence (IBTR) and contralateral breast tumor recurrence (CBTR) [6, 7]. Therefore, it is important to estimate the risk of IBTR and CBTR based on the surgically resected DCIS specimens and the clinical characteristics of patients.

When a partial resection is performed for a patient with DCIS, IBTR may occur even if complete resection is achieved; a previous study found that the 5- and 10-years local recurrence rates were 8.3–9.6 and 12.7–15.4 % when local irradiation was included in treatment, and 16.6–20.7 and 20.0–30.5 % when local irradiation was not included, respectively [8–13]. In Japanese patients, when both invasive carcinoma and DCIS were combined, the 10-year IBTR rates were reported to be 8.5 % after partial resection plus irradiation and 17.2 % after partial resection alone [14]. Positive surgical margins and the absence of local irradiation have been established as significant risk factors for IBTR in DCIS patients treated with partial resection. On the other hand, a 10-year IBTR rate after partial resection for DCIS was reported to be only 3.3 % after surgical therapy alone in a study conducted in a Japanese institute that treated a large number of patients [15].

A mastectomy should prevent IBTR, but CBTR may still occur, with reported 5- and 10-years CBTR rates of 3.3–3.6 and 6.9–7.9 %, respectively [9, 12]. In the National Surgical Adjuvant Breast and Bowel Project (NSABP) B-24 study, CBTR was shown to occur more often in patients with estrogen receptor (ER)-positive DCIS than in those with ER-negative DCIS (8.9 vs. 5.6 %) [8], suggesting that the expression of hormone receptors (HRs) by DCIS may predict subsequent contralateral breast cancer [16]. However, risk factors for CBTR are not well established in Japanese DCIS patients.

In this study, we examined the IBTR and CBTR rates after surgical therapy for primary DCIS in a cohort of Japanese patients. In order to identify risk factors for IBTR and CBTR, we compared these recurrence rates between subclasses of DCIS with various clinicopathological features.

## Patients and methods

### Patients

Of 5,731 consecutive patients who received treatment for primary breast carcinoma in the National Cancer Center Hospital (NCCH), Tokyo, between 1993 and 2008, 353 patients (6.1 %) were histologically diagnosed with DCIS. Four patients were excluded from the cohort because clinical, pathological, or immunohistochemical data were not available. Because the purpose of this study included a risk evaluation of CBTR, we also excluded 48 patients who received bilateral total or partial breast resections to synchronous or metachronous bilateral breast cancers: 23 had synchronous bilateral breast cancers, and 25 had past contralateral breast cancer when they underwent surgery to treat the existing DCIS. The pathological diagnoses in the 25 past contralateral breast cancers were invasive ductal carcinoma in 14, DCIS in 7, lobular carcinoma in situ (LCIS) in 2 and unknown in 2. Only 10 of these 25 patients underwent systemic therapy, which consisted of chemotherapy in 3 cases, endocrine therapy in 1 case, and both chemotherapy and endocrine therapy in 1 case. The type of systemic therapy used for the remaining 5 patients is unknown.

Among the remaining 301 patients, partial breast resections (lumpectomy or quadrantectomy) and total mastectomies were initially performed in 173 cases and 128 cases, respectively. For all cases in which partial resection was considered, intraoperative frozen section diagnosis of surgical margins were performed. When the margins were tumor-positive, additional resections were performed until the margins were shown to be negative. In 38 patients, the planned operation was changed from a partial resection to a total mastectomy because of a positive surgical margin. Whole, surgically resected specimens were cut into tissue blocks and processed to give permanent formalin-fixed paraffin-embedded sections. In the remaining 135 patients who finally received partial mastectomy, surgical margins were found to be positive (<1 mm from the tumor) in the permanent sections from 48 patients. Of these, 13 patients chose to undergo a subsequent total mastectomy, 12 patients underwent an additional partial resection, where surgical margins turned out to be negative (1 mm or more from the tumor), 17 patients underwent radiotherapy without additional breast resection; and 6 patients refused an additional resection or irradiation. Thus in total, 122 patients underwent a partial mastectomy (of whom 95 were treated using irradiation to the residual ipsilateral breast), and 179 patients underwent a total mastectomy.

Ninety-one patients underwent axillary lymph node dissection, and 124 patients underwent sentinel node biopsies, and lymph node metastasis was not detected in any of these 215 patients. The remaining 86 patients were not assessed using axillary staging. Twenty-six patients also underwent adjuvant systemic endocrine therapy, but the remaining 275 patients were not administered an adjuvant therapy.

The median follow-up time was 2,106 days (range 30–6,530 days). Follow-up was performed every 6 months for the first 5 years following surgery, and then every year for a further 5 years. Most patients were examined by tumor palpation, mammography, and ultrasonography. Further diagnostic imaging was performed depending on the symptoms or concerns of the patients. We reviewed the following clinicopathological features of 301 cases using the medical charts: age, menopausal status, body mass index (BMI), family history (FH) of breast cancer, tumor size, and surgical margin status of partially resected specimens. This study was approved by the internal review board of the National Cancer Center.

### Histology and immunohistochemistry

Histopathological characteristics of hematoxylin-eosin (HE)-stained slides of the representative cut surface of the main tumor were reviewed by two observers (N.T. and H.T.) with regard to histological subtype and nuclear grade (NG). In 4 cases, histological diagnosis was changed from DCIS to microinvasive carcinoma, with invasion foci <1.0 mm in diameter. The histological subtypes of DCIS were classified into 7 groups: comedo, cribriform, solid, papillary, and low papillary according to the predominant histological pattern [17], as well as solid-papillary, flat, and LCIS. The solid-papillary subtype was immunohistochemically confirmed to show neuroendocrine differentiation using anti-synaptophysin and anti-chromogranin A antibodies (Dako, Carpinteria, CA, USA). NG was evaluated according to the criteria of the Consensus Conference Committee [17].

HR expression status, including ER and progesterone receptor (PR), and human epidermal growth factor receptor 2 (HER2) were re-examined immunohistochemically using the representative cut surface of formalin-fixed paraffin-embedded tissue sections from each of the 301 tumors. ER and PR were assayed using a mouse anti-ER monoclonal antibody (clone 1D5, Dako) and a mouse anti-PR monoclonal antibody (clone PgR636, Dako), respectively, on a Dako autostainer according to the manufacturer’s instructions. HR positivity was evaluated using the Allred score system, and a score of 3 or more was judged as positive, whereas a score of 2 or less was judged as negative [30]. For HER2 detection, the HercepTest (Dako) was used according to the manufacturer’s instructions, and a score of 2+ or 3+ was judged as HER2-positive [31]. The surrogate intrinsic subtype was classified as follows: HR+/HER2−, HR+/HER2+, HR−/HER2+, and HR−/HER2−.

### Statistical analysis

The distribution of different parameters between patients was compared using the Chi squared test or Fisher exact test. Age, BMI and tumor size were numerical factors so we tried to decide cut-off values from each mean of distribution. BMI and tumor size showed log-normal distribution, but age cannot showed one peak distribution so we used median. The predictive value of parameters for IBTR and CBTR was calculated using the Cox univariate and multivariate proportional hazard general linear models. For the parameters that included a subgroup with no events, Cox analysis was not performed. The calculation was performed using JMP^®^9.02 software (SAS 2009).

## Results

### Clinicopathological characteristics

The median age of the 301 patients with DCIS was 50 years (range 26–83 years), 164 of these women were premenopausal, and the other 137 were postmenopausal. The median BMI was 21.5 (range 12.8–33.3). Forty-six patients (15 %) had a FH of breast cancer, which involved a first degree relative in 17 cases and a second degree or more distant relative in 29 cases. The median tumor size was 2.7 cm (range 0.1–9.5 cm) (Table 1).

**Table 1:** Clinicopathological characteristics of 301 ductal carcinoma in situ cases

Parameter	Number of cases (%)
Median age (range) (years)	50 (26–83)
Menopausal status	
Premenopausal	164 (54)
Postmenopausal	137 (46)
Median body mass index (range)	21.5 (12.8–33.3)
Family history	
No	255 (85)
Yes	46 (15)
1st degree	17 (6)
2nd degree	27 (8)
Unknown	2 (1)
Median tumor size (range) (cm)	2.7 (0.1–9.5)
Histological subtype	
Comedo	60 (20)
Non comedo	241 (80)
Cribriform	94 (31)
Solid	48 (16)
Papillary	45 (15)
Solid papillary	16 (5)
Low papillary	28 (9)
Lobular in situ	10 (3)
Necrosis	
Positive	72 (24)
Negative	229 (76)
NG	
NG1	181 (60)
NG2	56 (19)
NG3	64 (21)
HR status	
Positive	238 (79)
Negative	63 (21)
HER2	
Score 0 or 1+	244 (81)
Score 3+ or 2+	57 (19)
Surrogate intrinsic subtype (HR/HER2)	
HR+/HER2−	222 (74)
HR+/HER2+	12 (4)
HR−/HER2+	45 (15)
HR−/HER2−	22 (7)
Treatment	
Local treatment	
Total mastectomy	179 (59)
Lumpectomy	122 (41)
Lumpectomy + irradiation	95 (32)
Lumpectomy only	27 (9)
Surgical margin status	
Negative	99 (33)
Positive	23 (7)
Systemic treatment	
Tamoxifen only	26 (10)
None	275 (90)

*HER2* human epidermal growth factor receptor 2, *HR* hormone receptor, *NG* nuclear grade

Sixty cases (20 %) were of the comedo subtype, and 241 cases (80 %) were of the non-comedo subtype. Amongst the former, 94 (31 %) were cribriform, 48 (16 %) were solid, 45 (15 %) were papillary, 16 (5 %) were solid papillary, 28 (9 %) were low papillary, and 10 (3 %) were LCIS. Both the comedo and non-comedo subtypes (for example cribriform tumors) frequently showed signs of necrosis; the main tumor was necrotic in a total of 72 cases (24 %), 60 of which were the comedo subtype and 12 were the cribriform subtype. The NG was 1 in 181 tumors (60 %), 2 in 56 tumors (19 %), and 3 in 64 tumors (21 %).

The tumors were found to be HR positive in 238 cases (79 %) and negative in 63 cases (21 %), and tumors were HER2 positive in 57 cases (19 %) and negative in 244 cases (81 %). HR+/HER2−, HR+/HER2+, HR−/HER2+, and HR−/HER2− tumors were found in 222 (74 %), 12 (4 %), 45 (15 %), and 22 cases (7 %), respectively.

HR-tumor positivity was significantly associated with age 40 years or younger (90 vs. 77 % in the reference, *p* = 0.04), premenopausal status (89 vs. 67 % in the reference, *p* < 0.0001), smaller tumor size (<1.3 cm) (88 vs. 76 % in the reference, *p* = 0.03), non-comedo subtype tumor (88 vs. 43 % in the reference, *p* < 0.0001), absence of necrosis (90 vs. 44 % in the reference, *p* < 0.0001), and lower NG (NG1) (93 vs. 58 % in the reference, *p* < 0.0001). In contrast, HER2 status was associated or tended to be associated with postmenopausal status (23 vs. 15 % in the reference, *p* = 0.07), larger tumor size (1.3 cm or larger) (21 vs. 12 % in the reference, *p* = 0.08), comedo subtype tumors (55 vs. 10 % in the reference, *p* < 0.0001), presence of necrosis (50 vs. 9 % in the reference, *p* < 0.0001), and higher NG (NG2 or NG3) (38 vs. 6 % in the reference, *p* < 0.0001) (Table 2).

**Table 2:** Relationship between hormone receptor and HER2 status and the clinicopathological characteristics of ductal carcinoma in situ patients

Parameter	Total cases	No. of cases (%) HR-positive	*p*	No. of cases (%) HER2-positive	*p*
1. Age (years)					
≤40	41	37 (90)	*0.04*	7 (17)	0.74
>40	260	201 (77)		50 (19)	
2. Menopausal status					
Premenopausal	164	146 (89)	<*0.0001*	25 (15)	*0.07*
Postmenopausal	137	92 (67)		32 (23)	
3. Body mass index					
≥22	132	108 (82)	0.27	23 (17)	0.52
<22	167	128 (77)		34 (20)	
4. Family history					
Yes	46	40 (87)	0.13	6 (13)	0.25
No	255	198 (77)		51 (20)	
5. Tumor size (cm)					
<1.3	73	64 (88)	*0.03*	9 (12)	*0.08*
≥1.3	228	174 (76)		48 (21)	
6. Microinvasion by review					
No	297	235 (79)	0.84	57 (19)	0.19
Yes	4	3 (75)		0 (0)	
7. Histological subtype					
Non-comedo	241	212 (88)	<*0.0001*	25 (10)	<*0.0001*
Comedo	60	26 (43)		33 (55)	
8. Necrosis					
Negative	229	206 (90)	<*0.0001*	21 (9)	<*0.0001*
Positive	72	32 (44)		36 (50)	
9. NG					
NG1	181	159 (93)	<*0.0001*	11 (6)	<*0.0001*
NG2, NG3	120	69 (58)		46 (38)	

*HER2* human epidermal growth factor 2, *HR* hormone receptor, *NG* nuclear grade

### Prognosis of DCIS

Amongst the 122 patients who underwent lumpectomy, 7 (5.7 %) suffered IBTR. The period between surgery and the diagnosis of IBTR in these 7 cases were 431, 799, 1,066, 1,298, 1440, 1960 and 4,054 days (median 1,575 days). Recurrent lesions were invasive ductal carcinoma in 3 cases, mucinous carcinoma in 2 cases, and DCIS in 2 cases. In 2 patients who were found to have a positive surgical margin during resection of DCIS and subsequently experienced IBTR, it was not determined whether these locally relapsed lesions were true recurrences or a secondary primary tumor. The IBTR rate was higher or tended to be higher in patients aged 40 years or younger [hazard ratio, 7.2 compared to the reference, using Cox univariate analysis, 95 % confidence interval (CI), 1.3–40.2, *p* = 0.03] and in those with lower NG (NG1) (8 vs. 0 % in the reference, *p* = 0.06 by Fisher exact test). A lack of radiotherapy and a positive surgical margin were not significantly associated with IBTR (6 vs. 4 % in the reference, *p* = 0.7; and 9 vs. 5 % in the reference, *p* = 0.8, respectively) (Table 3). Because the only significant risk factor for IBTR in the univariate analyses was younger age, multivariate analyses were not preformed.

**Table 3:**
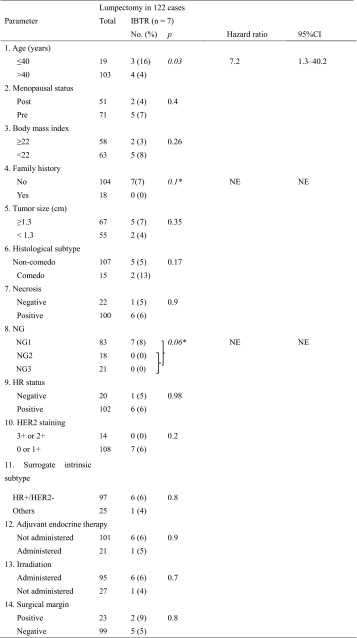
Cox univariate analyses to estimate the risk of clinicopathological parameters for IBTR amongst ductal carcinoma in situ patients who underwent lumpectomy

*CI* confidence interval, *HER2* human epidermal growth factor receptor 2, *HR* hormone receptor, *IBTR* ipsilateral breast tumor recurrence, *NE* not evaluable, *NG* nuclear grade

* The *p* value for differences in NG was calculated using Fisher’s exact test because no event occurred in the NG2/NG3 group

Amongst all 301 cases, CBTR occurred in 18 cases (6.0 %), 12 of which involved invasive ductal carcinomas and 6 involved DCIS. The median period of CBTR among these 18 cases was 2,579 days (range 1,206–5,182 days), after surgery for DCIS. The CBTR rate was higher or tended to be higher in patients with a FH of breast cancer (hazard ratio 3.0 by Cox univariate analysis; 95 % CI 1.0–7.9; *p* = 0.05), HR-positivity (7.6 vs. 0 % in the reference, *p* = 0.003 by Fisher exact test), and HR+/HER2− subtype tumors (hazard ratio, 5.6 by Cox univariate analysis; 95 % CI 1.1–101; *p* = 0.03) (Table 4). An HR+/HER2− tumor was an independent risk factor for CBTR on multivariate analysis (hazard ratio 5.1; 95 % CI 1.0–92.6; *p* = 0.04) (Table 5).

**Table 4:**
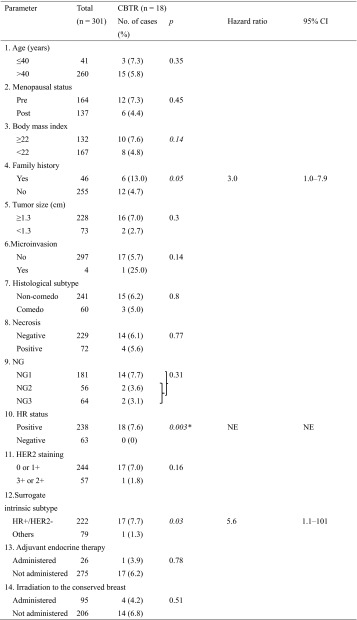
Cox univariate analyses to estimate the risk of clinicopathological parameters for CBTR in ductal carcinoma in situ patients

*CBTR* contralateral breast tumor recurrence, *CI* confidence interval, *HER2* human epidermal growth factor receptor 2, *HR* hormone receptor, *NE* not evaluable, *NG* nuclear grade

* The *p* value for difference in HR status was calculated using Fisher’s exact test because no event occurred in the HR-negative group

**Table 5:** Cox multivariate analysis to identify clinicopathological parameters that are independent risk factors for CBTR in ductal carcinoma in situ patients

Parameter	Total (*n* = 301)	CBTR (*n* = 18)			
		No. of cases (%)	*p*	Hazard ratio	95 % CI
Surrogate intrinsic subtype					
HR+/HER2−	222	17 (7.7)	*0.04*	5.1	1.0–92.6
Others	79	1 (1.3)			
Family history					
Yes	46	6 (13.0)	*0.07*	2.7	0.9–7.2
No	255	12 (4.7)			

*CBTR* contralateral breast tumor recurrence, *HER2* human epidermal growth factor receptor 2, *HR* hormone receptor, *CI* confidence interval

## Discussion

Due to the development of a nationwide screening program using imaging and needle biopsy techniques, the age-adjusted incidence of DCIS has increased 1.5 fold over the last 20 years [based on the data of the Surveillance, Epidemiology, and End Result Program (SEER) of the National Cancer Institute] [1]. Because DCIS is a precursor of invasive and potentially metastatic disease, understanding the relationship between clinicopathological characteristics and prognosis would help to clarify the natural history of cancer development and to establish methods for the secondary prevention of invasive breast cancer.

IBTR has been shown to be relatively common after breast-conserving therapy for DCIS, and the identification of risk factors for IBTR has been considered an important goal. In a meta-analysis, a comedo subtype tumor, necrosis, positive margin status, high tumor grade, and larger tumor size were significant risk factors for IBTR, and several studies showed that HR−HER2+ tumors might also be a risk factor [18, 19]. Available risk evaluation systems for local recurrence after partial resection for DCIS are the Van Nuys prognostic index (VNPI) and the multigene assay. In VNPI, risk is based on tumor size, necrosis, margin status, and age, with the latter being the only host factor [7, 20–22]. A multigene assay for DCIS can also identify DCIS patients with a higher risk of IBTR [23–25].

Our findings differ from those reported by a Japanese large-volume center, in which the IBTR rate after partial resection for DCIS without irradiation was very low, with a 10-year IBTR rate of only 3.3 % [15]. We found that the IBTR rate of patients who underwent partial resection for DCIS was 5.6 %. Ninety-nine of the 122 patients who underwent partial resection were found to have negative surgical margins by both intraoperative and permanent histological examinations. When intraoperative frozen section diagnosis revealed a positive surgical margin, additional resections were performed, and if this failed to give a negative margin (*n* = 23), irradiation was usually also performed (*n* = 19).

In the present study, an age of 40 years or younger and a lower NG were significant or nearly significant risk factors for IBTR by univariate analyses, whereas irradiation, surgical margin status, and tumor size were not significant risk factors for IBTR. Amongst patients treated at the NCCH, the low incidence of IBTR was probably due to the detailed histological examination of surgical margins, and the policy of always performing a complete surgical resection when it was possible. Further, multivariate analysis was not possible in this study due to the small number of events.

The identification of clinicopathological parameters that predict the risk of second primary tumors after DCIS might allow the earlier detection of CBTR. In this study, we found that HR+ and HR+/HER2− tumors, and a FH of breast cancer were significant risk factors for CBTR, and that a HR+/HER2− tumor was an independent risk factor in a multivariate analysis. These data support the results of other large-scale studies showing that a previous cancer and a FH of breast cancer could predict CBTR, and that non-invasive cancer carried the same level of relative risk for CBTR (8.1 vs. 11 %) as invasive cancer [6, 26, 27].

The NSABP B-24 study retrospectively evaluated the relationship between HR status and adjuvant endocrine treatment. In that study, adjuvant tamoxifen significantly reduced both IBTR and CBTR rates amongst ER-positive DCIS patients, but not ER-negative DCIS patients [8]. In addition, CBTR occurred more often in ER-positive DCIS patients than ER-negative DCIS patients (8.9 vs. 5.6 %), whereas CBTR rates after tamoxifen treatment were the same between these two patient groups [8]. Another study and a review showed that hormonal therapy for ER-positive breast cancer reduced the rates of IBTR and CBTR [16, 28, 29]. On the other hand, another milutcentre retrospective study from Japan showed that the incidence of CBTR per 1000 person-years was 5.1 without endocrine therapy and 3.6 among those with endocrine therapy in pTis and pT1mic patients [32]. Based on the results of the study we report here, HR positivity in DCIS may be a predictor for subsequent CBTR in Japanese patients. These findings justify a further study to clarify if chemoprevention using tamoxifen or an aromatase inhibitor can reduce the occurrence of CBTR in Japanese patients. However, a number of study limitations need to be considered. These are its retrospective nature, the inclusion of patients from only a single institution, and a median follow-up period of less than 10 years.

In conclusion, our findings show that the rate of IBTR in DCIS patients who underwent partial resection was higher in women aged 40 years or younger. HR+/HER2− tumors and a FH of breast cancer were risk factors for CBTR.
